# Inheritance and development of traditional martial arts under the perspective of group dynamics: a case study of Ma’s Tongbei martial arts

**DOI:** 10.3389/fpsyg.2025.1476857

**Published:** 2025-03-10

**Authors:** Weilei Yang, Yinwei Huang

**Affiliations:** ^1^School of Physical Education and Health, Guangdong Polytechnic Normal University, Guangzhou, China; ^2^School of Physical Education, Jinan University, Guangzhou, China

**Keywords:** group dynamics, martial arts, Ma’s Tongbei martial arts, group cohesion, sport psychology

## Abstract

**Introduction:**

In the modern context, the inheritance and development of traditional martial arts face numerous challenges. This study focuses on Ma’s Tongbei martial arts and aims to explore its inheritance and development from the perspective of group dynamics. It will reveal the deep-seated principles of the traditional martial arts inheritance mechanism and provide references and inspirations for modern martial arts education.

**Methods:**

This research mainly adopts the ethnographic research method to deeply analyze Ma’s Tongbei martial arts group and study its inheritance and development from the perspective of group dynamics.

**Results:**

The group structural characteristics of Ma’s Tongbei martial arts are analyzed, revealing the complex internal social relationships and norms within its unique social system, as well as the central position of the master-disciple relationship. It is found that group cohesion plays a crucial role in maintaining the stability of martial arts styles and promoting their development. It can inspire members to actively learn and inherit traditional skills and encourage innovation under the concept of “holistic integration and versatility in martial arts”, keeping Tongbei martial arts vibrant and adaptable to the needs of modern society.

**Discussion:**

Through the analysis of the group dynamics of Ma’s Tongbei martial arts, this study provides important references and inspirations for modern martial arts education. Meanwhile, potential future research directions are pointed out, including the study of martial arts group dynamics in cross-cultural contexts and the exploration of interactions and group dynamics between different martial arts styles, aiming to provide theoretical support and practical guidance for the modern inheritance of traditional martial arts.

## 1 Introduction

### 1.1 Traditional Chinese martial arts

There are numerous schools of traditional Chinese martial arts, among which Ma’s Tongbei martial arts is just one. For example, Shaolin wushu is one of the outstanding representatives of traditional Chinese martial arts, renowned worldwide for its rich history, unique skills, and profound cultural connotations. Chinese martial arts are rich in content and have significant stylistic differences. Some, such as Tai Chi, pursue the strategy of using softness to overcome rigidity. Their movements are slow and gentle, and they contain profound philosophical ideas and principles of health preservation (Lin et al., 2007). Others are renowned for their vigorous intensity, such as Nanquan (Southern Fist), which prioritizes explosive power and incorporates vocalization techniques (Ma, 2016). While schools like Baguazhang focus on agile footwork and dynamic body mechanics. Collectively, these diverse martial traditions form a rich tapestry of Chinese martial culture, each carrying unique historical, cultural, and philosophical significance.

However, these differences are more evident in terms of style and the expression of strength. From a broader perspective, many martial arts schools share numerous commonalities. In terms of their transmission methods, they all place great emphasis on master-apprentice lineages, featuring relatively clear inheritance systems (Wang, 2018). The older generation of martial artists passed on their exquisite skills and unique understanding of martial arts to the next generation through oral teaching and heartfelt instruction. This inheritance is not only a relay of techniques but also a transmission of martial arts culture and spirit. Martial arts etiquette is an important component of traditional Chinese culture and a moral code of conduct that all practitioners adhere to. Whether for those who practice or teach martial arts, etiquette is a part they highly value (Yu, 2014). Etiquette regulates the behavior of martial artists in apprenticeship ceremonies and daily practice and exchange activities, especially ritual movements such as the salute of clasping hands and bowing. All these actions show the martial artists’ respect for their teachers and martial arts. Their every word and deed also reflect the spirit of martial arts etiquette. The spirit of martial morality is the soul of martial arts. All schools emphasize that martial artists should possess justice, bravery, tenacity, and humility. They stand up against injustice, adhere to principles of fairness and justice in competition, and do not bully the weak with strength, which reflects the moral responsibility of martial artists. At the same time, each martial arts school has its community with common pursuits and goals, and they have a strong cohesion.

The cultural characteristics of Chinese martial arts in terms of cultural inheritance, etiquette standards, and the spirit of martial morality are not unique. Looking at the history of world martial arts development, Karate has not only established a lineage of historical inheritance but also emphasizes respect for the spirit of the martial way and has developed a rigorous etiquette procedure (Rielly, 2011). This cultural commonality is not only found in Karate but is also vividly reflected in traditional martial arts systems such as Taekwondo. Taekwondo focuses on politeness, integrity, perseverance, self-control, and an indomitable spirit, pursuing the dual cultivation of technique and spirit (Roesner, 2012). Therefore, they have all formed their unique cultural inheritance, etiquette standards, and the spirit of martial morality in their respective development processes, which essentially have similarities with Chinese martial arts.

The uniqueness of Ma’s Tongbei martial arts lies in its breakthrough of the limitations of traditional martial arts schools and its creation of an inclusive martial arts system. It not only inherits the traditional essence of Chinese martial arts but also extensively integrates the strengths of various schools, demonstrating an open and inclusive philosophy. This has injected powerful vitality and broad development potential into it. Additionally, Ma’s Tongbei not only promotes international exchanges but also absorbs the advantages of sports projects including fencing, budo, and taekwondo, mainly in terms of their training methods and competition rules, making it more diverse and rich. Therefore, taking Ma’s Tongbei martial arts as a research subject is of great significance and representativeness for revealing the inheritance and development of traditional martial arts in the contemporary era.

### 1.2 History of Ma’s Tongbei martial arts

Ma’s Tongbei martial arts is a part of traditional Chinese martial arts. It is not a specific sect, but rather a comprehensive martial arts system with a wide range of content. In the construction of the Tongbei martial arts system, Mr. Ma Fengtu is a central figure. Drawing upon extensive knowledge from various sources and throughout history, Mr. Ma adopted the philosophy of “Holistic integration and versatility in martial arts” to extract the strengths of different martial arts styles and to make connections between them. Through this approach, he gradually developed a well-rounded martial arts system (Ma, 1997).

However, during the process of transmission, due to historical changes and regional differences, the name of Tongbei martial arts may have been expressed in various ways, leading to a blurred understanding of its true essence. Nevertheless, Professor Ma Mingda holds a deep respect for and a sense of responsibility toward traditional martial arts, dedicating himself to the research of Tongbei martial arts. He delved into the works, techniques, and essence of past generations of practitioners, systematically organizing them, and thus established an accurate and comprehensive definition for Tongbei martial arts. This is evident in the article “From Tongbi to Tongbei - on origin of Tongbei wuxue” where it is stated, “Tongbei is not a specific style of martial arts, nor is it a traditional sect in the conventional sense. It is a category of martial arts, a comprehensive and well-structured martial arts system with extensive content. It can also be described as an integrated martial arts system that draws from the strengths of various sources and integrates them” (Ma, 2001). Based on his research and organization of Tongbei martial arts, Professor Ma Mingda has put forth a comprehensive and clear definition. His research goes beyond merely unifying the terminology associated with Tongbei martial arts; it has provided a deeper understanding and cognition of the system. This has laid a solid foundation for the continuation and development of Tongbei and has also set the right course for subsequent scholars and enthusiasts. Overall, the establishment of the concept of Ma’s Tongbei martial arts represents a reclamation of its intellectual identity, which to a certain extent has significantly enhanced its cohesion.

The Ma’s Tongbei martial arts system is primarily divided into two main parts: the theoretical system and the technical system, which are interdependent and complementary to each other. On the one hand, from the perspective of the theoretical system, Tongbei martial arts inherits the “Wen Tong Wu Bei” (Literary and martial skills are both well-prepared) ideology of the Yan and Li schools (Ma, 2000). Hence, among its disciples, there is a saying that “without reading 10,000 books, it’s difficult to become a true practitioner of Tongbei” highlighting the significance of literary cultivation. The foundation of the theoretical system lies in the inheritance of the “Wen Tong Wu Bei” ideology from the Yan and Li schools. This not only underscores Tongbei Martial Arts’ emphasis on learning and self-cultivation but also highlights the close connection between martial skills and wisdom in life. Teaching the students of Tongbei martial arts, it is emphasized that only by possessing extensive knowledge and profound cultural understanding can one truly become proficient in Tongbei and better fulfill the broader goal of serving society.

Ma’s Tongbei martial arts revolves around the core concept of “holistic integration and versatility in martial arts.” It suggests choosing the strengths of various schools based on sound reasoning, rather than haphazardly assembling without basis, and it discourages stagnant and closed-minded approaches. “Holistic integration and versatility in martial arts” hold profound significance, emphasizing the “fusion of ancient and modern wisdom, and the harmonization of internal and external cultivation.” The concept of “integrating ancient and modern knowledge” involves drawing wisdom from the history timeline. Traditional martial arts embody the refined techniques and philosophical essence passed down through generations of predecessors. The classic techniques, training methods honed over centuries, and insights into martial philosophy represent invaluable cultural treasures. Meanwhile, modern martial arts have evolved through advancements in scientific training and competitive frameworks. By merging historical martial principles with contemporary methodologies, practitioners can both preserve the essence of traditional martial arts and leverage modern scientific innovations to enhance training efficacy.

“The concept of ‘harmonizing internal and external cultivation’ emphasizes the comprehensive integration of multiple strengths. It not only requires practitioners to meticulously refine external martial skills—such as mastering techniques, and routines, and flexibly applying combat strategies to demonstrate exceptional martial prowess—but also places paramount importance on nurturing internal virtues, including martial ethics and mental discipline. Furthermore, this philosophy encompasses the assimilation of essential elements from diverse martial styles and schools, incorporating their strengths into one’s practice to achieve well-rounded development.” The concept of “drawing on the best from different martial arts styles” embodies the core philosophy of “holistic integration and versatility in martial arts.” In the field of martial arts, different styles and regional practices each have their unique characteristics. For instance, Tai Chi emphasizes yielding to force with softness, remaining still while in motion, and focusing on health and harmony (Horwood, 2008). By adopting the strengths of various schools and integrating these advantages into one’s martial practice, a more comprehensive, distinctive style can be developed, which can then leverage greater strengths.

Within the framework of the “holistic integration and versatility” concept, Ma’s Tongbei martial arts has developed the concept of “Tongbei Jin” (Tongbei Power). “Tongbei Jin” unifies the four jin techniques of “swallowing and spitting”, “opening and closing,” “rising and falling,” and “twisting and turning” into a cohesive whole. From a technical perspective, “Tongbei Jin” primarily draws from the power principles of the Piguaquan style. It comprehensively incorporates the distinctive power characteristics of other schools such as Bajiquan, Fanziquan, and Chuojiao, resulting in a unique pattern of power circulation and a distinctive martial arts style (Ma, 2001).

On the other hand, from a technical perspective, Ma’s Tongbei martial arts primarily encompasses two major aspects: weaponry and boxing techniques. In daily training, especially during the initial learning phase, the foundation is usually established through boxing techniques. The introductory arts of Ma’s Tongbei martial arts system include “Tongbei Tantui” (Ma, 2011), “Baji Xiao Jia,” and “Twelve Strikes.” “Baji Xiao Jia” is akin to the regular script in calligraphy, where each move and posture is executed calmly and composedly, totaling 24 movements, including initiating and concluding postures. The main purpose of “Baji Xiao Jia” is to prepare for practicing “Bajiquan” and to explore the expression of “Tongbei Jin” ultimately transitioning into the practice of the larger frame techniques (Ma, 2014). From here, it can be seen that the progression within Ma’s Tongbei martial arts system is characterized by distinct stages, especially evident in the transition from one technique to another. “Twelve Strikes,” as an introduction to the system, consists of 12 techniques, that serve as routine training in daily practice (Chen and Feng, 2023). In addition to the foundational techniques, Ma’s Tongbei system also encompasses various series of techniques, including the “Piguaquan” series, the “Bajiquan” series, the “Fanziquan” series, and the “Weapons” series. Among these, the “Piguaquan” series forms the cornerstone of Tongbei system. Importantly, the system preserves many classical martial arts treasures within its weaponry, such as the “Po Feng Dao” and the “Bian Gan” techniques, among others.

It’s essential to clarify that, like any cultural form dominated by physical techniques, the techniques within Tongbei martial arts system are not entirely original creations. Instead, they are a combination of inherited and original elements, developed under the principle of “integrating the best from various sources” These techniques are then integrated with the distinctive practice style of “Tongbei Jin, “thus preserving the integrity of the original techniques while infusing them with new vitality. The construction of Tongbei martial arts system is built upon a complete foundation of both theoretical and technical aspects. Through generations of Ma’s lineage’s transmission and dedication, the Ma’s Tongbei Martial Arts system has gained acclaim both domestically and internationally, garnering a considerable number of followers.

### 1.3 Group dynamics and sport

Eys et al. explore dynamic group environments in sport and exercise, highlighting the importance of group dynamics in physical activity and reviewing progress on a number of key and ongoing topics as well as emerging ones. Recommendations for future research are made in order to advance the field. Understanding group dynamics requires consideration of the prevalence of groups in sports and exercise environments, as well as the fulfillment of basic needs such as a sense of belonging. The article also identifies four key research areas: team environments, structural issues, team processes and emergencies, and applied principles of team dynamics. They suggest that there are tremendous opportunities for researchers to contribute to the theory, research, and practice of group dynamics in sports and exercise (Eys et al., 2019).

Research related to group dynamics and sports has also provided insights into the interactions within sports teams and explored how these interactions can be optimized to improve overall team performance and individual athlete development. Research suggests that coaching behaviors (including leadership, fairness, and competence) are critical in shaping team cohesion and reducing internal conflict. These attributes of the coach can influence team members’ role clarity, role conflict, and thus overall team effectiveness (González-Ponce et al., 2022). Group cohesion is recognized as a key factor in improving individual athlete skills and reducing negative experiences. A cohesive team promotes positive member development in personal and social skills, initiative, cognitive skills, and goal setting (Bruner et al., 2014a). Group norms are standards that guide team behavior and expectations, and they are influenced by both individual and social factors. Understanding the formation and role of group norms can help sports teams build more favorable team environments, particularly in competition, practice, and social settings (Bruner et al., 2014b). Cohesion is considered to be a remarkable attribute of successful teams, whether in the work, military, sports, or exercise fields. It plays such an important role in group dynamics that some social scientists call it the most important small-group variable (Burke et al., 2014).

The presence of cliques in sports teams is inevitable, and they have a complex impact on team dynamics and performance. Cliques can lead to isolation and conflict, but they can also promote better teamwork and individual performance through inclusive behaviors and positive team management strategies (Martin et al., 2015). In addition, organizational culture and group dynamics are closely linked and interact in sports. Establishing a strong and coherent organizational culture helps to support the positive development of young athletes and creates and maintains positive group dynamics through the role of cultural leadership (Storm et al., 2020).

In summary, the above studies have highlighted the importance of understanding and managing group dynamics in sports, particularly in terms of promoting positive athlete development, enhancing team performance, and reducing internal conflict. To achieve these goals, sports organizations and coaches need to focus on optimizing group structure, enhancing cohesion, clarifying norms, managing small groups appropriately, and fostering a positive organizational culture. However, there is a gap in research on group dynamics in martial arts. In this study, Ma’s Tongbei martial arts is selected as a case study, and based on the theory of group dynamics, its role in the inheritance and development of traditional martial arts is discussed in depth, with a view to providing a new perspective for the inheritance and dissemination of traditional martial arts.

## 2 Theoretical foundations

Group dynamics is a social psychological theory devoted to exploring the developmental patterns of groups, their internal dynamics, and their relationships with individuals, other groups, and society. Lewin (1947) published an article in the inaugural issue of the journal Interpersonal Relations entitled ‘Frontiers in group dynamics. Concept, method, and reality in social science. In this article, Lewin pays particular attention to participation in social change and emphasizes that “in social research, the experimenter must take into account factors such as the personality of individual members” (Beauchamp et al., 2007).

Group dynamics suggests that groups are much more effective than single individuals because a common set of social goals and values develops within the group, and these standards bind the members together, prompting individual motivation to combine with the group’s goals. It is easier to trigger change by changing the group than by changing the perceptions or behavior of individuals alone. As long as the group’s values remain the same, it is difficult for individuals to abandon the group’s standards (Miao et al., 2000). Since the beginning of social psychology, group dynamics has been widely regarded as one of the core areas of the discipline. The term “group dynamics” has a double meaning: on the one hand, it describes the intrinsic dynamics and evolutionary properties of groups; on the other hand, it represents a field of science that focuses on the study of group behavior. Generally speaking, group dynamics is not regarded as a separate discipline, but rather as an important field of study under social psychology and sociology (Widmeyer et al., 2002).

Groups significantly influence the psychology and behavior of individuals. Individuals, after joining a group, will have common psychological changes such as identity, belonging, role consciousness, overall consciousness, and exclusion consciousness, which constitute the psychological characteristics of the group. At the same time, the behaviors of group members will also show common trends of change, such as facilitation, inertia, standard, herd, and deindividuation behaviors, and these common changes constitute the behavioral characteristics of the group. By comparing group decision-making with individual decision-making, we can gain a deeper understanding of the unique manifestations of group psychology and behavior in the decision-making process (Zhao, 2021).

The importance of group dynamics theory in exploring martial arts is mainly reflected in the fact that it provides a framework for understanding how martial arts groups interact with each other through factors such as social structure, cohesion, role distribution, and behavioral changes to jointly promote the transmission and development of martial arts skills. Considering that martial arts is not only about the learning of techniques, but also about the transmission of culture and spirituality, the group dynamics perspective can help us to analyze in depth how these elements are manifested in the master-disciple relationship, the interactions between practicing partners, and how they affect the behavioral patterns of the individuals and the group, thus providing scientific theoretical support and practical guidance for the teaching, dissemination and preservation of martial arts.

## 3 Research methodology

Research design: This study primarily employs ethnographic research methods, taking Ma’s Tongbei martial arts as a specific case. In its regular intensive training venues, in-depth and systematic investigations were carried out. The research period spanned from 2020 to 2024, during which the researchers conducted a total of 600 h of field participation and observation, truly integrating into the learning and practical environment of Ma’s Tongbei. During the research process, the researchers adopted case study methods and combined them with cross-referencing of literature, aiming to gain a comprehensive and multi-angle understanding of the research subjects. As participants, the researchers engaged firsthand in the daily training, communication, and inheritance activities of Tongbei martial arts, striving to reveal the internal mechanisms of martial arts inheritance and development from within the group. Through this direct participation and observation, the purpose of the study is to understand the interactions between individuals and groups in the process of martial arts learning, and how these interactions promote skill transmission and innovation.

Data collection: This study will collect data through three main avenues: participant observation, personal journaling, and deep reflection. In terms of participant observation, the researchers will immerse themselves in the learning environment of Ma’s Tongbei martial arts. Besides directly participating in learning Tongbei martial arts, will also join the Tongbei WeChat group, carefully documenting the communication methods, collaboration mechanisms, and role differentiation among group members, focusing on the behavioral performances and interaction patterns of the group during the learning process, providing abundant empirical materials for analyzing group dynamics.

Personal journaling, on the other hand, serves as an in-depth excavation of the individual learning experience of the participants, covering various stages from initially joining Ma’s Tongbei martial arts to gradually delving deeper. It records the difficulties and challenges, breakthroughs and progress, emotional fluctuations, and insights gained during the learning process, comprehensively presenting the individual’s growth trajectory and psychological journey within the group learning atmosphere.

In the deep reflection stage, the researchers will systematically analyze the group dynamic data obtained from participant observation and the individual change content in personal journals. By applying relevant theoretical frameworks and academic perspectives, they will delve into the intrinsic mechanisms and interrelationships of factors such as group cohesion, influence among members, and recognition of collective goals in the inheritance and development of Ma’s Tongbei martial arts. In doing so, they aim to construct a comprehensive understanding system of the multi-level and multi-dimensional dynamics effect of Tongbei martial arts groups.

Data analysis: This study employs a scientifically rigorous approach to encode practical notes and observation records, accurately refining key themes and patterns of group interaction, learning motivation, and individual growth. As both practitioners and researchers, to minimize the influence of subjectivity on the research, critical thinking is always maintained during the data analysis and interpretation process to avoid over-interpretation and biased explanations. When analyzing, an objective stance is upheld, focusing on exploring the shaping effect of groups on individual learning paths, as well as the specific ways in which individuals promote skill acquisition and cultural inheritance through group interactions. Special attention is paid to group dynamic factors that affect the inheritance and innovation of Ma’s Tongbei martial arts, such as cohesion, communication modes, decision-making participation, and member relationships. These factors are deeply analyzed for their promoting or hindering effects, thereby comprehensively revealing the core role and internal logic of group dynamics in the inheritance and development of Tongbei martial arts. This provides new theoretical and practical insights into the inheritance of traditional martial arts.

## 4 Discussion and analysis

Group Structure deals with the way groups are formed, their membership, and their interrelationships in group activities. These structural factors not only define the behavioral patterns of group members but also help us to understand and predict group actions and their effectiveness. Group structure is therefore critical to understanding group behavior and improving group performance. In studying group structure, key variables include leadership style, size, social norms, role allocation, member status, and overall membership composition (Zhao, 2021).

As a unique school of traditional Chinese martial arts, the inheritance and development of Ma’s Tongbei martial arts not only relies on its superb skills and profound cultural heritage but is also inextricably linked to its unique group structure and group dynamics. In the process of participating in the training of Ma’s Tongbei martial arts, we have personally experienced the unique charm of its group structure and gained a deeper understanding of the inheritance mechanism of Tongbei martial arts. In the following section, from the perspective of group dynamics, combining knowledge of Tongbei martial arts and the author’s personal experience, we will delve into the characteristics of the group structure of Ma’s Tongbei martial arts, especially its strong cohesive force, and how this cohesive force has contributed to the continued development of this martial arts school.

### 4.1 Structure of the Ma’s Tongbei martial arts community

The group structure of Ma’s Tongbei martial arts is not a simple collection of individuals, but rather a social system that contains complex social relationships and norms, and whose internal members interact with each other to shape the dynamics of the school’s transmission and development. Within this system, the master-disciple relationship is central, and elements such as role allocation, group norms, and collective decision-making mechanisms together form a multi-layered social structure that provides a solid organizational foundation for the transmission and development of Tongbei martial arts.

The mode of transmission of traditional martial arts mainly includes the forms of family transmission and master-disciple transmission. However, with the increasing development of society, the limitations of family-based transmission have gradually emerged and its influence is relatively limited. Therefore, the master-disciple relationship is the core of Ma’s Tongbei martial arts transmission and the cornerstone that maintains the stability of the group structure. The master is not only the transmitter of skills but also the transmitter of culture, values, and behavioral norms. This relationship goes beyond the mere transmission of knowledge but is also a spiritual inheritance that builds a strong identity and emotional bond. The master’s words and teachings will subtly influence the disciple’s martial arts philosophy, moral cultivation, and behavior, and shape their martial arts personality (Ma, 2003). Ma’s Tongbei martial arts emphasizes “strictness in the selection of students, strictness in the transmission of skills,” and masters will examine the character, qualifications, and understanding of martial arts when accepting students to ensure that skills are passed on to those who are truly passionate about martial arts and have a good character (Ma, 2014). This strict screening mechanism ensures the quality of the transmission of Tongbei martial arts and strengthens the emotional bond between master and disciple. The master-disciple relationship is not only about teaching skills but also about emotional exchange and spiritual inheritance. The care and teaching of the master to the apprentice, and the respect and gratitude of the apprentice to the master, together constitute a warm and powerful emotional atmosphere within the Tongbei martial arts community. This close master-disciple relationship is also in line with the contextual learning theory proposed by Lave and Wenger (1991). The importance of learning through an apprenticeship in a community of practice is emphasized. Through teaching by word and example, the master passes on martial arts skills, cultural concepts, and values to the apprentice by osmosis, so that the apprentice gradually integrates into the cultural atmosphere of Tongbei martial arts and develops a sense of identity and belonging to the school.

Upon first entering Tongbei martial arts, beginners are struck by Master’s rigor and attention to etiquette. Master not only teaches martial arts skills but also focuses on cultivating the character and cultivation of beginners. The principle of “cultivating morality before practicing martial arts,” and the principle that martial arts should be based on morality and should not be used as a bullying tactic, has instilled in the beginner a deep sense of respect and love for the Tongbei martial arts. Mr. Ma Fengtu, the founder of Ma’s Tongbei martial arts, attached great importance to the master-disciple relationship. He believed that the inheritance of martial arts is not only the teaching of techniques but more importantly the inheritance of spirit. He once said, “Martial arts is not just a technique, it’s a discipline” (Ma, 2003). Mr. Ma Fengtu’s son, Professor Ma Mingda, has also inherited his father’s belief that there must be certain requirements and conditions for accepting disciples and that the quality of apprenticeship should be improved rather than the number of disciples. It can be seen that a quality master-disciple relationship is the “lifeline” of the Tongbei martial arts and is the key to ensuring that the school can be passed on from one generation to the next. In addition, the establishment of a master-disciple relationship also implies a responsibility and commitment. The master promises to teach his life’s work and to guide his pupil’s growth, while the pupil promises to respect his master and study hard to pass on and develop the Tongbei martial arts. This two-way responsibility and commitment further strengthen the emotional bond between master and apprentice, making the master-apprentice relationship an important guarantee for the inheritance and development of Tongbei martial arts, forming a united, friendly, and loving Tongbei family.

Clear role allocation and hierarchical order are the keys to maintaining stability, promoting inheritance, and building harmonious group relations. There are clear role assignments within the group, such as master, senior disciple, junior disciple, etc., and each role corresponds to different responsibilities and obligations. This hierarchical order is not to suppress individuals but is an important mechanism to maintain the stability of the group and orderly inheritance (Zhao, 2021). As an assistant to the master, the senior disciple assumes the responsibility of guiding the junior disciple and passing on his experience, as well as being a role model for the junior disciple to follow. The junior, on the other hand, has to respect both the master and the senior disciple, learn with an open mind, and train hard. This clear distribution of roles and hierarchical order creates a benign interaction that promotes the effective transmission of skills and harmonious development within the group. Obviously, this hierarchical order is not rigid and stereotypical but is based on mutual respect and trust. Senior disciple guidance to his Junior is not condescending, but rather, he uses his own experience and realization to help his junior to better understand and master the martial arts techniques. Junior disciple respect for Seniors is not blind obedience, but recognition of experience and ability. This harmonious relationship is an important manifestation of unity and love within the Tongbei martial arts community. The distribution of roles and hierarchical order in Tongbei martial arts reflects the ethical concept of “order of seniority” in traditional Chinese culture and is also in line with the role theory of group dynamics (Biddle, 2013). Role theory suggests that individuals play different roles in groups, each with a corresponding set of behavioral norms and expectations. Clearly defined role allocation and hierarchical order help to regulate the behavior of members, reduce conflict, and increase the efficiency of the group. In Ma’s Tongbei martial arts, this kind of role allocation and hierarchical order is not only conducive to the transmission of skills but also to the transmission of martial virtues. In the process of instructing his junior, a senior disciple should not only teach techniques but also set a good example for his junior and guide him to develop good martial virtues.

Strict group norms and etiquette are an important guarantee for shaping the culture of Tongbei martial arts, strengthening identity, and passing on the spirit of martial virtues. Norms and etiquette such as respect for teachers, humility, and courtesy are an important part of the Ma’s Tongbei martial arts, which strengthens the members’ sense of responsibility and belonging and shapes the group’s cultural atmosphere (Ma, 2018). Tongbei martial arts focuses on etiquette, from the start to the finish, every movement implies reverence for the martial arts and respect for the tradition (Ma, 2014). Members also treat each other with courtesy, reflecting the cultivation of martial virtues and friendship toward fellow members. These norms and etiquettes not only regulate the behavior of the members but more importantly reinforce their identity and make them members of the Tongbei martial arts cultural community. These norms and etiquette are not empty forms but are the embodiment of the spirit of Tongbei martial arts. Honoring teachers reflects respect for knowledge and experience, humility and courtesy reflect the cultivation of martial virtues, and these spiritual qualities are an important safeguard for the inheritance and development of Tongbei martial arts. In addition, the Tongbei group norms and etiquette also reflect Tongbei Wushu’s inheritance of traditional culture and its emphasis on martial virtues. Martial Arts is the soul of martial arts and is the moral quality and spiritual realm that martial arts practitioners should possess. Through strict group norms and etiquette, Tongbei martial arts integrates the spirit of Wude into daily training and life, cultivates the moral integrity of its members, and shapes their Martial Arts personalities. For example, Ma’s Tongbei martial arts requires the practitioner to have a “calm mind and upright body” during practice. This is not only a technical requirement, but also a moral one, requiring the practitioner to maintain inner peace and integrity, and not to be impatient or bullying. The importance of these norms and etiquette is deeply appreciated in the personal practice of Tongbei martial arts. Before the start of each training session, the practitioner salutes the master as a sign of respect and appreciation. When sparring with senior disciples and junior disciples, they also have to be courteous to each other and point to point, so as not to hurt each other. These etiquette norms not only make the training more standardized but also teach the Tongbei disciples how to respect others more and improve their martial virtues.

In addition, the group dynamics demonstrated in Ma’s Tongbei Martial Arts Age have a role in shaping behavior and stimulating potential as a means of promoting individual growth. Phenomena such as herd behavior, social comparison, and de-individuation in group interactions influence members’ training attitudes, learning outcomes, and behavioral patterns, reflecting the role of group dynamics in shaping individual behavior. In group training, generalist disciples will observe, learn from, and compare with each other, and this social comparison will motivate them to train harder and improve their skills. At the same time, the group also creates certain norms and pressures that motivate members to be disciplined and train seriously. This group dynamics can stimulate the potential of individuals and promote their growth and progress. Thus, group dynamics are an important manifestation of the vibrancy within the Tongbei martial arts community and its ability to continue to cultivate excellence and promote the development of martial arts techniques. Festinger’s (1954) Social Comparison Theory explains this phenomenon well, emphasizing that individuals assess their own abilities and values by comparing themselves with others. In the Tongbei martial arts community, social comparisons and mutual incentives among members promote the continuous improvement of their skills and martial virtues.

### 4.2 Cohesion of the Ma’s Tongbei martial arts community

The Ma’s Tongbei martial arts group demonstrates strong cohesion, which is a key factor in maintaining the stability of the group and promoting the development of the martial arts school. The cohesion of Tongbei martial arts mainly stems from the shared history, traditions, and values among its members, the close master-disciple relationship and fellowship, the common training goals and pursuits, as well as the sense of ritual and belonging in group activities.

Members’ identification with the history, culture, and values of Ma’s Tongbei martial arts forms a common belief and goal that can enhance the centripetal force of the group (Ma and Ma, 1995). Tongbei martial arts have a long history and deep cultural heritage, and its concept of “holistic integration and versatility,” the idea of “integration of reason and image, body and use,” and the spirit of “once you get the art, you must test the enemy” have all deeply influenced every generation of Tongbei practitioners. The spirit of combat has deeply influenced every generation of Tongbei practitioners. This common cultural identity has given them a strong sense of belonging and mission, and they are jointly committed to passing on and developing Tongbei martial arts. This common cultural identity is the cornerstone of the cohesion of Tongbei martial arts, which brings together members from different backgrounds and of different ages to form a cultural community with common goals and values. In the Tongbei martial arts community, the collective consciousness is reflected in the members’ recognition of the history, culture, and values of Tongbei martial arts, as well as their shared sense of responsibility for the inheritance and development of Tongbei martial arts. Tongbei martial arts emphasizes the importance of “holistic integration and versatility,” and advocates learning from the strengths of various martial arts styles and adopting the advantages of all (Ma, 2001). This requires members of Tongbei martial arts to have an open mind and a spirit of tolerance, which is also an important part of the culture of it. Tongbei martial arts is not a closed and conservative martial arts school, but one that actively absorbs the advantages of other martial arts schools and constantly innovates and develops. This spirit of openness and tolerance makes followers appreciate Tongbei martial arts even more and strengthens their determination to pass it on and carry it forward.

The emotional bond between masters and disciples and the mutual support among fellow disciples can build a solid network of social relationships and enhance members’ sense of belonging and responsibility (Huang et al., 2021). Within the Tongbei martial arts community, there is a deep sense of camaraderie between masters and disciples and between fellow disciples, who respect, help, and encourage each other to overcome difficulties and make progress together. This close network of social relationships provides members with emotional support and strength in action, enabling them to pursue their martial arts dreams with greater determination. The atmosphere of solidarity and appreciation in Tongbei martial arts is an important manifestation of the cohesion of the Tongbei martial arts group, which enables the members to form a strong sense of trust and responsibility and to work together to safeguard the interests and honor of the group. Social exchange theory (Homans, 1958) can explain this mutually beneficial social relationship by emphasizing that individuals seek to maximize rewards and minimize costs within groups of people. In the Tongbei martial arts community, members support and help each other and work together to safeguard the interests of the group, reflecting the principle of reciprocity in social exchange theory. Tongbei martial arts groups often organize activities where Tongbei disciples train together, study together, and live together, experiencing the joys of growth and the trials of frustration together, and this shared experience makes the bond between followers even deeper.

The members’ common pursuit of martial arts skills and their understanding of “Tongbei Jin” form a common goal and promote cooperation and mutual motivation among members. The martial art of Tongbei pursues the realm of comprehensive advancement and all-around development, members continue to refine their skills by sparring and discussing with each other during training. This common pursuit has enabled them to form a positive learning atmosphere, stimulating their potential and promoting the continuous development of their martial arts skills. The common pursuit of martial arts skills is an important manifestation of the cohesion of Tongbei martial arts, which has led to the formation of a healthy competitive relationship among the members, and the joint promotion of the progress of martial arts skills. Goal-setting theory (Locke and Latham, 2002) emphasizes the importance of clear, challenging, and achievable goals in motivating individual and group performance. In the Tongbei martial arts community, members share the pursuit of martial arts skill refinement, and this common goal motivates them to strive for excellence. The motivational effect of the common goal motivates each individual to aspire to improve his or her martial arts skills and to achieve the state of “Tongshen Dahua” (A realm of transcendence, freedom, and omnipotence; Ma, 2001).

Joint participation in training, competitions, and ceremonial activities enhances the collective consciousness and emotional experience of members and strengthens group identity. The Tongbei martial arts group organizes regular training activities. Training is not only a platform for improving skills but also an opportunity for members to exchange feelings and enhance friendships. By participating in these activities together, members feel more deeply the cultural charm of Tongbei martial arts and the power of the group, strengthening their identity and sense of belonging. Group activities are an important manifestation of the cohesion of Tongbei martial arts, which enables members to form common memories and emotional experiences, and enhances the vitality and centripetal force of the group. Durkheim’s theory of fundamental forms of religious life emphasizes the role of rituals and symbols in constructing collective consciousness and social solidarity (Durkheim, 2016). In the Tongbei martial arts community, various ritual activities strengthened members’ collective consciousness and enhanced their sense of identity and belonging to the Tongbei martial arts.

### 4.3 Group dynamics

The cohesion of the Ma’s Tongbei martial arts community is not static but rather translates into a driving force for the development of the school. The cohesion of Tongbei martial arts motivates its members to actively learn and pass on the traditional techniques while encouraging innovation under the concept of “holistic integration and versatility” to maintain the vitality of the martial arts. Tongbei martial arts is not static, but rather evolves and innovates as it is passed on. While learning traditional techniques, members also actively explore new techniques and training methods, combining Tongbei martial arts with the needs of modern society to make it more adaptable to the times. The spirit of inheritance and innovation is an important reason why Tongbei martial arts has been able to maintain its vitality throughout its long history.

The atmosphere of learning and mutual support within the group is conducive to the cultivation of excellence and ensures the continued development of the Ma’s Tongbei martial arts school. The Tongbei martial arts community focuses on talent development, with masters providing personalized guidance to their apprentices according to their characteristics, and senior brothers helping their apprentices to solve their learning difficulties. This good learning atmosphere and mutual support provide fertile soil for the growth of talents, which makes Tongbei have many talents and successors. This talent cultivation mechanism is an important guarantee for the continuous development and growth of Tongbei martial arts.

Cohesion enhances members’ sense of identity and pride in Tongbei martial arts, prompting them to actively participate in the dissemination and promotion of martial arts culture. Members of Tongbei Martial Arts Academy actively participate in various martial arts competitions and performances to show the glamor of Tongbei Martial Arts Academy to society and spread the spirit of Tongbei. This active cultural dissemination has expanded the influence of Tongbei martial arts and provided a broader space for its development and growth. In the face of the challenges of the external environment, cohesion enables the group to unite and work together to cope with difficulties and maintain the stable development of the martial arts school (Du and Wang, 2021). In modern society, traditional martial arts faces challenges from the impact of Western sports culture and social transformation. With its strong cohesion, the Tongbei martial arts community is able to unite as one and respond positively to these challenges, innovate and develop on the basis of inherited traditions, and maintain the vitality and viability of the martial arts school. This spirit of unity and solidarity is the key to Tongbei Wushu’s ability to survive and develop in the face of adversity. In the 1980s, there was a wave of “de-traditionalisation” in the Chinese martial arts community, with some believing that traditional Martial Arts had become obsolete and should be replaced by modern competitive Wushu. In the face of this challenge, the Tongbei martial arts community adhered to its philosophy did not blindly follow the trend, and ultimately succeeded in preserving the traditional characteristics of Tongbei martial arts.

## 5 Conclusion

Through in-depth analyses of the group structure of Ma’s Tongbei martial arts and its strong cohesive force, this study reveals the key factors that have enabled this traditional martial arts school to maintain its unique style and sustain its development over time. Group dynamics played a crucial role in the transmission and development of Ma’s Tongbei martial arts. This demonstration of group wisdom and collective power not only provides a new perspective for understanding the inheritance mechanism of traditional martial arts but also offers valuable lessons for the practice and theory of modern martial arts education.

In conclusion, the case of Ma’s Tongbei martial arts provides us with a framework for understanding how traditional martial arts can survive and develop in modern society. By strengthening community building, enhancing cohesion, and innovating on the basis of inheritance, the Ma’s Tongbei martial arts will not only be able to adapt to the needs of the times but also make an important contribution to the revival of Chinese martial arts. Future research should continue to explore how group dynamics can be utilized to promote the transmission and innovation of martial arts in different cultural and social environments, in order to ensure the vitality and continuity of this ancient art form.

## Data Availability

The original contributions presented in the study are included in the article/supplementary material, further inquiries can be directed to the corresponding author/s.
